# FOURTH AGEISM AND THE TECHNOLOGICAL IMAGINARY OF LONG-TERM CARE

**DOI:** 10.1093/geroni/igad104.1602

**Published:** 2023-12-21

**Authors:** Paul Higgs, Chris Gilleard

**Affiliations:** University College London, London, England, United Kingdom; University College London, London, England, United Kingdom

## Abstract

Many high-income countries are pre-occupied with the rising number of older people needing long term care and the consequent demand for a labour force to carry out these tasks. In many other sectors of the economy the problem of labour supply is increasingly being met by the introduction of sophisticated technology. Seeking to extend this strategy to the long-term care sector (LTC) has led to research and development in various care technologies. This paper examines the imaginaries fuelling such efforts, as distinct from the outcomes of such research. One such imaginary is that of a fourth age, a state of frailty and dependency that requires others to carry out instrumental and related activities of daily living to those deemed to have fallen under its shadow. This creates a set of contrasting goals, between on the one hand the desire to deliver LTC in a way that maximises the dignity and integrity of the persons receiving LTC and on the other the desire to reduce demands on the workforce. While the former stresses the maximisation of the humanity of LTC the latter is often motivated by the reduction of the (human) labour inputs involved. These dilemmas will arguably become acute if such various technological imaginaries become realities. This paper argues that forms of implicit fourth ageism present in current thinking need to be challenged at the conceptual stage of this technological imaginary.

